# The pathway of ligand entry from the membrane bilayer to a lipid G protein-coupled receptor

**DOI:** 10.1038/srep22639

**Published:** 2016-03-04

**Authors:** Nathaniel Stanley, Leonardo Pardo, Gianni De Fabritiis

**Affiliations:** 1 Computational Biophysics Laboratory (GRIB-IMIM), Universitat Pompeu Fabra, Barcelona Biomedical Research Park (PRBB), C/Doctor Aiguader 88, 08003 Barcelona, Spain; 2 Laboratori de Medicina Computacional, Unitat de Bioestadística, Facultat de Medicina, Universitat Autònoma de Barcelona, 08193 Bellaterra, Spain; 3 Institució Catalana de Recerca i Estudis Avançats, Passeig Lluis Companys 23, 08010 Barcelona, Spain

## Abstract

The binding process through the membrane bilayer of lipid-like ligands to a protein target is an important but poorly explored recognition process at the atomic level. In this work we succeeded in resolving the binding of the lipid inhibitor ML056 to the sphingosine-1-phosphate receptor 1 (S1P_1_R) using unbiased molecular dynamics simulations with an aggregate sampling of over 800 μs. The binding pathway is a multi-stage process consisting of the ligand diffusing in the bilayer leaflet to contact a “membrane vestibule” at the top of TM 7, subsequently moving from this lipid-facing vestibule to the orthosteric binding cavity through a channel formed by TMs 1 and 7 and the N-terminal of the receptor. Unfolding of the N-terminal alpha-helix increases the volume of the channel upon ligand entry, helping to reach the crystallographic pose that also corresponds to the predicted favorable pose. The relaxation timescales of the binding process show that the binding of the ligand to the “membrane vestibule” is the rate-limiting step in the multi microseconds timescale. We comment on the significance and parallels of the binding process in the context of other binding studies.

Over 60% of currently marketed drugs target membrane proteins^1^. These proteins are targeted so frequently because they mediate a large variety of cellular responses to extracellular signals, and are often linked to disease. The initial event of these cellular responses is the binding of ligands or drugs to a target membrane protein. Despite its importance in the process, measuring the binding interactions in membrane proteins is substantially more difficult than for water-soluble proteins and remains a challenge^2^. Unbiased molecular dynamics (MD) simulations in the microsecond timescale have recently been used to yield novel insights into the processes of ligand binding^3^,^4^,^5^. However, in these cases, the ligand progressed from the extracellular aqueous environment to the apolar binding site crevice at the transmembrane (TM) domain of the membrane protein.

The situation is substantially complicated when the ligand is itself a lipid. In the case of phospholipid transport across cellular membranes, such as via P4-ATPases or ATP binding cassette (ABC) transporters, ligand entry into the binding site occurs from the membrane bilayer^6^. In addition, membrane phospholipids can be metabolized upon cell activation into potent lysophospholipid mediators, such as lysophosphatidic acid (LPA) and sphingosine 1-phosphate (S1P). These bioactive lipid mediators regulate diverse processes such as angiogenesis, cardiac development, neuronal survival, and immunity via the interaction with the G protein-coupled receptors (GPCRs) LPA_1_-LPA_6_Rs and S1P_1_-S1P_5_Rs, respectively^7^. The recent crystal structure of S1P_1_R in complex with the ML056 inhibitor (a.k.a. W146) (Fig. 1a)^8^ has shown that, despite S1P_1_R having a molecular architecture similar to the other members of the GPCR family^9^, the ligand access to the binding site likely occurs from the lipid bilayer via an opening between TM helices, in marked contrast to other GPCRs.

In this work, we try to understand the binding process of a lipid mediator to the receptor using large scale unbiased all-atom MD simulations on the GPUGRID^10^ distributed computing infrastructure. Other studies have previously used simulations to investigate the binding of ligands to GPCRs^4^,^11^,^12^ from the solvent environment. However, the quantitative binding reconstruction of the endogenous ligand via the lipid bilayer was never possible due to the reduced sampling available^13^. Here, thanks to large-scale molecular simulations of over 800 microseconds, we have been able to characterize the binding of the lipid ligand to its crystal-bound pose, as well as to produce a quantitative description of major steps along the binding pathway. Finally, we highlight the role of a flexible N-terminal helix and water in the binding process. All data is available upon request to the authors.

## Results

### Computational binding experiment

**Figure 1:**
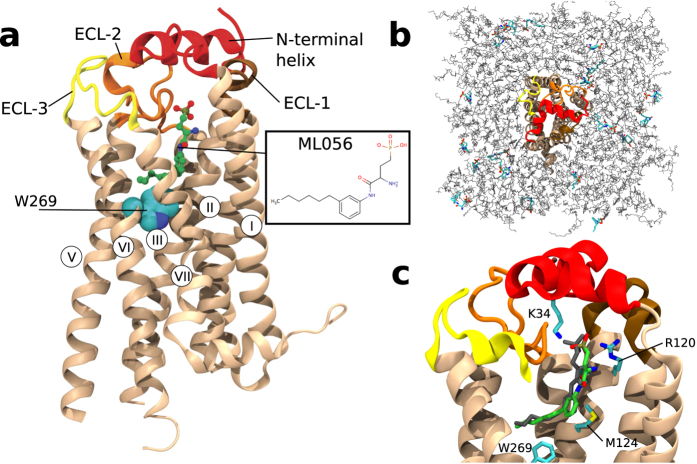
The structure of S1P_1_R and the simulation setup. (**a**) Crystal structure of S1P_1_R (PDB id: 3V2Y). (**b**) Top-down perspective of starting structures for MD simulations. S1P_1_R shown in cartoon representation in red. Top and bottom lipid layer shown in gray. ML056 ligands shown as thick sticks colored in cyan, blue, red and gold. Water, ions, and hydrogens are not shown for clarity. (**c**) Closest bound pose of the ML056 ligand from these simulations (green) relative to the crystal structure (gray), along with several key residues (cyan). The pose pictured has an RMSD of <1 Å relative to the crystal-bound pose.

We inserted 19 ML056 inhibitors spread roughly evenly between both leaflets of a lipid membrane containing palmitoyl-oleoyl phosphatidylcholine lipids (POPC), cholesterol, and S1P_1_R (see methods and Fig. 1b). Three different rounds of MD simulations, with an aggregate sampling over 800 microseconds, were performed (Table 1).

In the first series of simulations (I1 in Table 1), none of the ML056 inhibitors spontaneously bound the S1P_1_R binding site, but in seven cases the ligand remains bound to the surface of the receptor, mainly to amino acids in TMs 1 and 7 (six simulations, see below) and TMs 1 and 2 (one simulation). Next, fifty simulations were spawned from each of these seven surface-bound structures in order to progressively sample the binding event (R1 in Table 1). Notably, in 60% of these subsequent simulations there was a clear progression into the S1P_1_R orthosteric binding pocket located within the TM helical bundle (RMSD < 12 Å relative to the crystal bound pose), in 28% of the simulations ML056 remained surface-bound, and in 12% of the simulations the ligand moved back into the bulk membrane. For consistency, we have only respawned simulations if several similar events have previously occurred to ensure that the dominant pathway is correctly sampled as done in automated adaptive sampling schemes^14^,^15^ (see also the Methods section for more detail).

Thus, a final series of 500 simulations (R2 in Table 1) were spawned from 28 trajectories that reach the TM binding pocket with ligand RMSD < 5 Å relative to the crystal bound pose^8^. In 82% of these simulations, ML056 remained below RMSD values of 5 Å, resulting in an average RMSD-to-bound of 3.6 Å. The simulation with the lowest RMSD value (<2 Å) relative to the crystal bound pose is shown in Fig. 1c, which is slightly above to the average fluctuation seen in a simulation started from the crystal bound pose (see Supplementary Fig. 2). This computationally-derived binding pose reproduced the main contacts between ML056 and the receptor observed in the crystal structure: the ionic interactions between the zwitterionic head group of the ligand and R120^3.28^ and E121^3.29^, and the hydrophobic interactions between the acyl chain of the ligand and an hydrophobic/aromatic pocket in TMs 3 and 6 mainly formed by M124^3.32^, F125^3.33^, L128^3.36^, L272^6.51^ and F273^6.52^.

### The pathway of ligand entry

**Figure 2:**
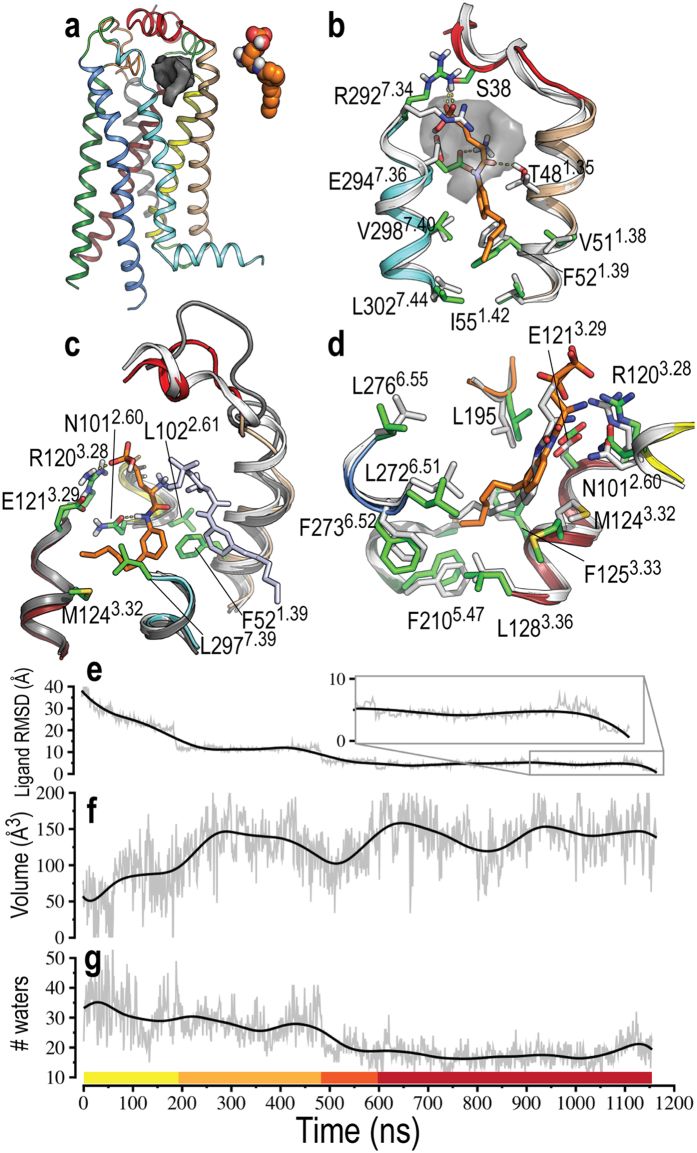
The pathway of ligand entry. The binding of ML056 to S1P_1_R can be subdivided into four steps (panels (**a**–**d**)). (**a**) ML056 (in orange spheres) diffuses from the bulk of the membrane toward an entrance channel (gray surface) in S1P_1_R (TM 1: wheat, TM 2: yellow, TM 3: dark red, TM 4: gray, TM 5: green, TM 6: blue, TM 7/helix 8: light blue, Nterm: red, ECL 2: orange). The simulation time of the ligand in this step is shown as a yellow bar in panel (**g**). (**b**) The polar head group of ML056 (orange sticks) is attracted to a “membrane vestibule” by the interaction of S38, T48^1.35^, R292^7.34^ and E294^7.36^ (green sticks). The hydrophobic tail of the ligand is accommodated in a hydrophobic pocket between TMs 1 and 7. The entrance channel is shown as a transparent gray surface and the crystal structure of S1P_1_R is shown in white for comparison. The simulation time for this step is shown as an orange bar in panel (**g**). (**c**) ML056 (orange sticks) moves from the “membrane vestibule” (shown in light blue sticks for comparison purposes) to the entrance channel. The crystal structure of S1P_1_R and an earlier snapshot, in which the N-terminal helix is unfolded due to ligand entering the channel, are shown in white and gray, respectively. The simulation time for this step is shown as a dark orange bar in panel (**g**). (**d**) In the final step, ML056 enters the orthosteric binding cavity from the channel. The computationally-derived binding pose (orange and green sticks for ML056 and S1P_1_R, respectively) reproduced the main contacts observed in the crystal structure (white helices and sticks). The simulation time for this step is shown as a red bar in panel (**g**). (**e**) RMSD values of ML056, relative to the bound-pose observed in the crystal structure, along the MD trajectory. (**f**) Volume of the cavity (gray surface in panels (**a**,**b**)), forming the entrance channel, along the MD trajectory as calculated with POVME^18^. (**g**) Number of water molecules within 5 Å of ML056 along the MD trajectory.

In contrast to β-adrenergic and muscarinic receptors (and most other GPCRs), in which the binding site is fully accessible from the extracellular environment, the N-terminus (in red in Fig. 1) and ECL2 (in orange) of S1P_1_R buries the binding site from the extracellular environment in a similar manner as rhodopsin. The entrance/exit channels for retinal in rhodopsin have been proposed to occur through the lipid bilayer, via two openings located between TMs 1 and 7, and TMs 5 and 6^16^,^17^. Thus, we aim to identify for S1P_1_R the pathway that connects the buried orthosteric binding pocket to the membrane bilayer. We explored pockets and channels from conformations obtained in the MD trajectories that account for the intrinsic flexibility of S1P_1_R^18^. The cavity located between TMs 1 and 7 is shown in Fig. 2a and the N-terminal domain that defines an entrance channel of the ligand is shown in Fig. 2b.

While the step-by-step interactions between ML056 and S1P_1_R were heterogeneous among trajectories, the binding pathway can be subdivided into four different stages (Fig. 2). An example of trajectory of binding is shown in supplementary Movie 1. ML056 moves slowly from bulk membrane to contact a lipid-facing cavity of S1P_1_R mainly formed by amino acids in TMs 1 and 7. In these conformations the phosphonate group of ML056 is attracted and captured by R292^7.34^ and S38, while the protonated primary amine interacts with E294^7.36^ (Fig. 2b). The end-terminal hydrophobic tail of the ligand accommodates in a hydrophobic pocket formed by V51^1.38^, F52^1.39^, I55^1.42^, V298^7.40^ and L302^7.44^. The ligand spent about 250 ns (Fig. 2g), interacting with these residues. The presence of a small cavity at the entrance of the orthosteric binding site has also been described in similar studies on β-adrenergic receptors^4^,^19^. In these cases, this cavity was named “extracellular vestibule”^4^ or “secondary binding site”^19^. Importantly, it was shown that ligands binding to this cavity in muscarinic receptors might act as allosteric modulators since they control the extent of receptor movement to govern a hierarchical order of G-protein coupling^20^.

In a third stage the ligand inserts the hydrophobic tail into this entrance channel to interact with L102^2.61^ and L297^7.39^. Eventually the zwitterionic head group of the ligand is moved from the “membrane vestibule” to the channel by a diverse set of polar interactions involving, in addition to R292^7.34^ and E294^7.36^, the side chains of D40, K41, or E42 in the N-terminal α-helix. However, it is important to note that these interactions are transient. Figure 2c shows a conformation in which the polar head group interacts with R120^3.28^ and N101^2.60^, whereas the hydrophobic tail interacts with F52^1.39^, M124^3.32^ and L297^7.39^.

In the final step of the binding process, ML056 enters the orthosteric binding cavity from the channel. The hydrophobic tail of the ligand enters the binding cavity before the polar head group. However, in a few cases the head went in first. In such cases we never saw a full binding event, and it is even possible that the ligand could leave the binding cavity and reentered tail-first. Once inside, the zwitterionic head group transiently interacts with R120^3.28^ and E121^3.29^, which are important to the bound pose (see above). Reaching the final stage requires the hydrophobic tail to pass between TMs 3 and 6 (sterically hindered by the bulky and centrally located M124^3.32^) and relocating the water molecules of the binding cavity. At this point the dominant motions are rearrangements of the head and tail such that all the favorable interactions can be formed (Fig. 2d).

### Ligand hydration and the role of water

Water molecules have a critical role in ligand binding processes^21^,^22^. We calculated the number of water molecules within 5 Å of the ligand in all the steps of the binding process (Fig. 2g). As ML056 moves progressively from the bulk membrane to the “membrane vestibule”, the number of waters molecules decreases from roughly 40 to 30. The entrance of the ligand into the channel and the orthosteric binding cavity further drops the number of water molecules to 15–20. While the number of bound water molecules does not change substantially inside the binding cavity, their position appears important. Because water molecules show a complex network of interactions with the key R120^3.28^ and E121^3.29^ residues and the incoming ligand, the competition between these transiently stable interactions relative to the native contacts seems to be a final barrier that must be overcome.

### Characterization of the binding process

**Figure 3:**
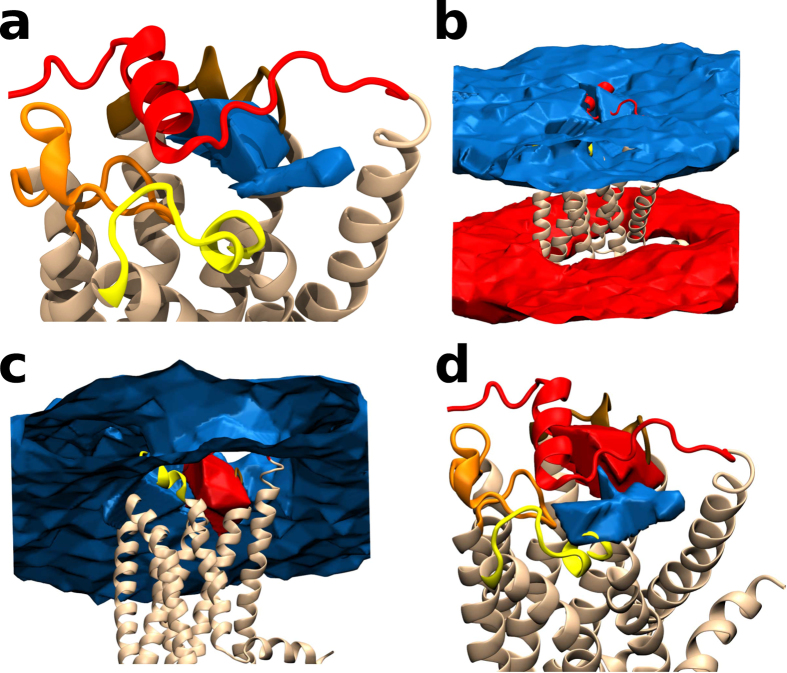
Free energy isosurface of the binding process. (**a**) The energy surface (blue) of the pathway of ligand entry from the “membrane vestibule” between TMs 1 and 7 and the orthosteric binding cavity. The isosurface is shown at 1 K_b_T above the minimum (the crystal-bound pose). (**b**) The MSM eigenvector structure shows the transitions occurring for the slowest process. Transitions are shown from one state (blue blob) to the other (red blob). The slowest relaxation is the flipping of ML056 between the bilayers (100 microseconds). (**c**) The transition from the bulk upper membrane leaflet to the “membrane vestibule” (1–10 microseconds). (**d**) The transition between the “membrane vestibule” and the bound state (less than 1 microsecond).

The fact that we have 19 undistinguishable ligand molecules in the membrane complicates a simple application of a Markov state model (MSM) analysis such as in^3^,^15^,^23^,^24^ for single ligands. Therefore, we use an alternative approach by computing the energetics of the ligand in a similar manner of refs 3,25 by reconstructing the energetic volumetric map of the ligand around the protein. We therefore built the equilibrium three dimensional occupancy map of the position of the phosphate group of the ligand (Fig. 3 and Methods).

The free energy isosurface shows the bound pose of the ligand as well as its binding pathway (Fig. 3a). The free energy minimum corresponds to the position of the phosphate as in the crystal bound pose. The pathway of entry is clearly shown to be between TMs 1 and 7. The population difference between the ligand being in the binding cavity and in the membrane bilayer is approximately 2 kcal/mol, i.e. at least an order of magnitude higher occupancy in the orthosteric binding volume. Note that the concentration of the ligand in the membrane is already high (10:5:1, POPC:cholesterol:ML056). The relaxation timescales of the slowest transitions in the system (Supplementary Fig. 1) show three separate processes and a well converged MSM. By analyzing the sign of eigenvectors of the MSM transition matrix these relaxations can be assigned to specific processes (see Methods). The slowest relaxation process can be identified with the flipping of the ligand between membrane leaflets (~100 μs) (Fig. 3b). The second slowest process is given by the transition from the upper leaflet to the “membrane vestibule” (Fig. 3c) between TMs 1 and 7 (1–10 μs). The third slowest process (Fig. 3d) is the transition from the “membrane vestibule” to the bound pose (~500 ns). As the leaflets flipping process is not important for binding, being both leaflets populated with the same amount of ligands, the second process of formation of the surface-bound structures (“membrane vestibule”) at the top of TMs 1 and 7 becomes the rate limiting step.

### The role of the N-terminal α-helix in ligand entry

**Figure 4:**
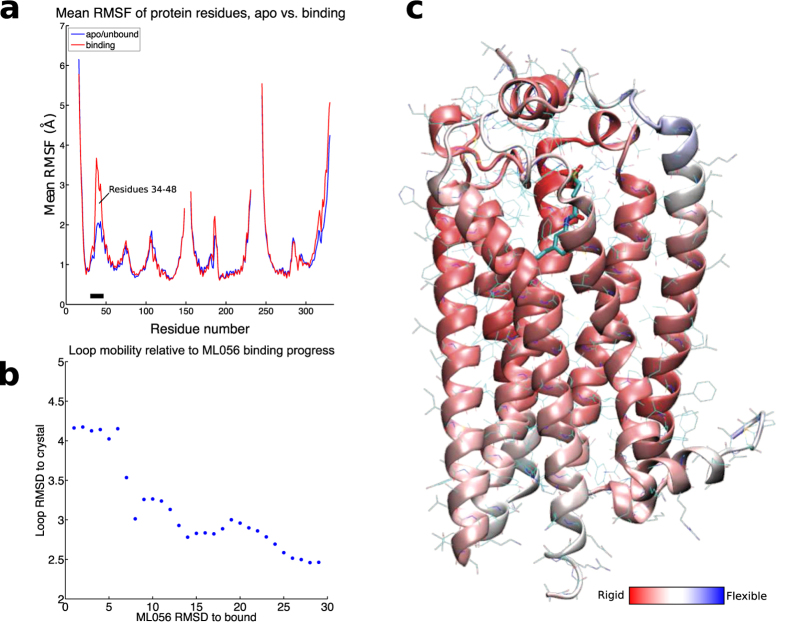
Flexible N-terminal helix allows access during binding. (**a**) Root mean square fluctuation (RMSF) of each residue of S1P_1_R. Blue line shows simulations where no ML056 was binding (>15 Å). Red curve shows RMSF for several trajectories during the binding process (<15 Å). A black bar is shown below residue 34–48. (**b**) RMSD of ML056 to bound versus the RMSD of the loop to its crystal structure. As ML056 binds, the loop becomes increasingly disordered. (**c**) S1P_1_R in cartoon representation colored by crystallographic B factor (a.k.a. temperature factor). Dark Red indicates rigid structure, dark blue indicates greater flexibility. Notably the N-terminal helix over the entrance is the most flexible part of the structure.

An interesting feature that was apparent in the various binding trajectories was the role of the α-helix of the N-terminal domain in defining the entrance channel of the ligand. The observed binding simulations suggest that ligand entry into this channel perturbs this N-terminal α-helix, causing partial unfolding (Fig. 2c) and the corresponding increase of the volume of the channel (Fig. 2f), which facilitates the entrance of the ligand into the orthosteric binding cavity. To probe this behavior, we show the RMSD of this α-helix relative to the conformation observed in the crystal structure against ligand RMSD relative to the crystal bound pose of all the 28 binding trajectories mentioned above (Fig. 4b, Supp. Movie 2). Clearly, as the ligand enters into the binding cavity (low values of RMSD) the conformation of the α-helix is more distant to the crystal structure (high values of RMSD). Importantly, in some trajectories this N-terminal domain refolds into the α-helix conformation seen in the crystal structure (Fig. 2c) once the ligand is located in the binding site apart from the entrance channel. In order to quantify the flexibility of this α-helix and assess its role in ligand entrance, we compared the average RMSF of the entire protein between the 28 respawned trajectories and the initial simulations that did not cause spontaneous ligand binding. We found a clear increase in average RMSF of residues 34 to 48 forming this α-helix (Fig. 4a). Furthermore, the beta factors of the crystal structure (Fig. 4c) show that this part of the N-terminus is inherently less stable than the rest of the protein, supporting the observation that the helix could undergo a helix-coil transition as shown in the simulations. Remarkably, this N-terminal helix of S1P_1_R is a loop in the recent crystal structure of LPA_1_R^26^. As the authors noted, this different conformation of this domain suggests that LPA_1_R preferentially receives ligands from the extracellular milieu, in contrast to membrane access for S1P_1_R ligands.

## Discussion

In this work we have shown the binding of a lipid-like ligand from the membrane bilayer directly to the orthosteric binding site of a GPCR using unbiased MD simulations. The ML056 inhibitor, studied here, binds S1P_1_R through a multi-step process that finally leads to the crystallographic binding pose. Our computer simulations have permitted to elucidate the binding pathway along with critical atomic interactions and conformational changes.

Similar to other studies of ligand binding to GPCRs^4^, there is a barrier to binding that occurs far away from the final binding pose. This barrier is formed by the interaction between the zwitterionic head group of the ligand and R292^7.34^ and E294^7.36^ in the “membrane vestibule” and the desolvation necessary for the ligand to enter the channel. Others have shown that for S1P binding, which has a similar zwitterionic head group to ML056, mutation of R292^7.34^ results in a drastic reduction in the EC50 for the receptor in whole cell experiments^27^, although the specific mechanism for this is unclear. This site could be a target for allosteric modulators, similar to locations recently identified on the muscarinic receptor^11^. In the final steps, the lipid tail fits snugly between hydrophobic and aromatic side chains in TMs 3 and 6, and the zwitterionic head group binds R120^3.28^ and E121^3.29^. A final barrier is due to rearrangements of the water molecules in the binding cavity and of dihedral angles in the head and tail of the ligand such that all these favorable interactions can be formed. Remarkably, in membrane-embedded ligands the favorable entropic desolvation of hydrophobic parts has already occurred, in contrast with lipid ligands that enter the binding cavity from the extracellular aqueous environment.

Along the binding process, the widening of the cavity by the flexible N-terminus and the desolvation of the ligand head group appear as general trends. However, the functional importance of the rearrangement of the N-terminal helix in binding also remains unclear. While it certainly needs to move somewhat in order for the ligand to be able to enter, its interactions with the ligand may be important to productive binding. Finally, it is unclear whether and what role residues along the binding pathway could play in allostery or biased signaling^11^,^28^,^29^. Residues such as those in the N-terminal helix or at the top of TM7 outlined here, could be targets for the design of such modulators.

## Methods

### System Preparation

The crystal structure of S1P_1_R was downloaded from the Protein Data bank (PDB: 3V2Y). Residues 1002 to 1161 were removed and uncharged terminal caps were placed at residues 231 and 245. The glucosamine attached to residue 30 was removed. The ML056 ligand was moved to its own file to aid in parameterization. The phosphate and amine of the ML056 ligand were assumed protonated, resulting in a zwitterionic, neutrally charged ligand. The receptor was then fed into the CHARMM-GUI^30^ online membrane building tool, where the receptor was automatically positioned in a 90 Å × 90 Å square membrane. A lipid membrane composed of 10:5:1 of POPC, Cholesterol, and a dummy lipid was used, with the dummy lipid later being mutated into ML056. The membrane was solvated with TIP3P water and 0.15 M NaCl. The system was rebuilt in VMD^31^ in order to generate a correct topology file, protonation and angles and dihedrals. CHARMM27 force field^32^ with CMAP terms^33^ was used for the protein. Lipids were parameterized with the CHARMM36 parameters, while the ParamChem^34^,^35^ web server was used to assign CGenFF^36^,^37^ parameters for the ML056 ligand. The ML056 molecules were situated >20 Å away from the receptor to minimize biased binding due to initial coordinates.

### Simulation protocol

All simulations were performed with ACEMD^38^ molecular dynamics software. The system was minimized for 500 steps, followed by equilibration in the NPT ensemble for 20 ns to allow the membrane to equilibrate. Frames were saved every 100 ps for all simulations. During the equilibration, restraints were placed on the heavy atoms of the protein and slowly relaxed over the first 5 ns, and the protein was allowed to move freely thereafter. The system was then simulated for 100 ns under the NVT ensemble. Structural snapshots were taken every 20 ns from this trajectory and used to spawn 200 simulations each on GPUGRID^10^, resulting in 1,000 simulations total. These simulations were ran for up to 1 microsecond each, resulting in an average length of 580 nanoseconds or 580 microseconds aggregate simulation. As these simulations ran, the ligand’s RMSD to the crystal bound pose was continuously monitored manually, and those with the lowest RMSD were inspected visually to discern if ML056 had begun interact with the receptor. In seven simulations, we saw that ML056 was below 15 Å RMSD to the crystal structure and appeared to interact with the receptor between either TM1 & TM7 (6 cases) or TM1 & TM2 (one case). We respawned 50 new simulations from snapshots from these seven trajectories, resulting in 350 new simulations. These simulations were run for an average length of 630 nanoseconds, giving additional aggregate sampling of 203 microseconds. A final structure was taken from the closest bound pose of these last runs in order to ensure the bound pose was stable and adequately sampled. A respawning round of 500 simulations of 100 ns each was sent, resulting in an additional 48 microseconds of aggregate simulation. For all cases of respawning, initial velocities for each simulation were reassigned based on velocities drawn from a Maxwell-Boltzmann distribution. The Langevin thermostat with a damping coefficient of 0.1/ps was used for all production simulations to maintain constant temperature for the simulations thereafter.

### Three-dimensional Markov state model

The master equation of a Markov process is built as


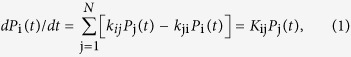


where *P*_i_(*t*) is the probability of state *i* at time *t*, and *k*_ij_ are the transition rates from *j* to *i*, and **K** = (*K*_ij_) is the rate matrix with elements *K*_ij_ = *k*_ij_ for *i* ≠ *j* and 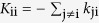. The equation d**P**/dt = **KP** has solution with initial condition **P**(0) given by **P**(*t*) = **T**(*t*)**P**(0), where we defined the transition probability matrix *T*_ij_(*t*) = (exp[**K***t*])_ij_ i.e. the probability of being in state *i* at time *t*, given that the system was in state *j* at time 0. *T*_ij_(Δ*t*) is estimated from the simulation trajectories for a given lag time Δ*t* using a maximum likelihood estimator compatible with detailed balance^39^. The eigenvector **π** with eigenvalue 1 of the matrix **T**(Δ*t*) corresponds to the stationary, equilibrium probability. Higher eigenvectors correspond to exponentially decaying relaxation modes^40^ for which the relaxation timescale is computed as 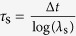, where *λ*_s_ is to the largest eigenvalue above 1. For long enough lag times Δ*t* the model will be Markovian.

A Markov state model of the system was built based on the three dimensional position of the ligand phosphate similarly to refs 3,40 using the HTMD software environment^41^. The phosphate coordinates of each lipid-ligand is treated independently and the data is discretized in space by using a simple regular space clustering algorithm and the discrete trajectories used to construct the MSM transition matrix *T*_ij_(t), giving the probabilities of jumps between clusters *i* and *j* at different lag times *t.* A maximum likelihood estimator was used to build the transition matrix which guarantees detailed balance. The relaxation timescales of the slowest processes were then computed (Supplementary Fig. 1). A lag time of 30 ns was finally used to construct the Markov model at which the relaxations are already into exponential behavior (flat in log-scale, see Supplementary Fig. 1).

### Hydration analysis

The hydration of the ligand and other residues was determined using VMD. The number of waters with an atom <5 Å from the residue of interest were counted and plotted over time.

### N-terminal helix flexibility analysis

To better understand the prevalence and significance of the flexibility in the N-terminal helix covering the binding pocket, we compared the mean RMSF of all residues between 28 trajectories in which the ligand bound <5 angstrom and all trajectories from the initial round of simulations (Fig. 4a). To further strengthen confidence in the trend seen, we plotted the RMSD of the loop to its crystal structure against the RMSD of the closest bound ML056 ligand (Fig. 4b).

## Additional Information

**How to cite this article**: Stanley, N. *et al*. The pathway of ligand entry from the membrane bilayer to a lipid G protein-coupled receptor. *Sci. Rep.* **6**, 22639; doi: 10.1038/srep22639 (2016).

## Supplementary Material

Supplementary Information

Supplementary Movie 1

Supplementary Movie 2

## Figures and Tables

**Table 1:** An overview of the different rounds of simulations performed in this work.

**Group ID**	**Description**	**Num. Starting Structures**	**Intended Num. of Simulations (runs × ns)**	**Aggregate simulation attained**
I1	Initial simulations	5	1,000 × 1,000	579 us
R1	Respawn, round one	7	350 × 1,000	204 us
R2	Respawn, round two	1	500 × 102	48 us

## References

[b1] Overington J. P., Al-Lazikani B. & Hopkins A. L. How many drug targets are there? *Nat. Rev*. D*rug Discov.* 5, 993–996 (2006). 17139284 10.1038/nrd2199

[b2] Wang W. . Label-free measuring and mapping of binding kinetics of membrane proteins in single living cells. Nat. Chem. 4, 846–853 (2012). 23000999 10.1038/nchem.1434 PMC3660014

[b3] Buch I., Giorgino T. & De Fabritiis G. Complete reconstruction of an enzyme-inhibitor binding process by molecular dynamics simulations. Proc. Natl. Acad. Sci. 108, 10184–10189 (2011). 21646537 10.1073/pnas.1103547108 PMC3121846

[b4] Dror R. O. . Pathway and mechanism of drug binding to G-protein-coupled receptors. Proc. Natl. Acad. Sci. 108, 13118–13123 (2011). 21778406 10.1073/pnas.1104614108 PMC3156183

[b5] Kruse A. C. . Structure and dynamics of the M3 muscarinic acetylcholine receptor. Nature 482, 552–556 (2012). 22358844 10.1038/nature10867 PMC3529910

[b6] Coleman J. A., Quazi F. & Molday R. S. Mammalian P4-ATPases and ABC transporters and their role in phospholipid transport. Biochim. Biophys. Acta BBA - Mol. Cell Biol. Lipids 1831, 555–574 (2013). 10.1016/j.bbalip.2012.10.006 PMC3562415 23103747

[b7] Chun J., Hla T., Lynch K. R., Spiegel S. & Moolenaar W. H. International Union of Basic and Clinical Pharmacology. LXXVIII. Lysophospholipid Receptor Nomenclature. Pharmacol. Rev. 62, 579–587 (2010). 21079037 10.1124/pr.110.003111 PMC2993255

[b8] Hanson M. A. . Crystal Structure of a Lipid G Protein–Coupled Receptor. Science 335, 851–855 (2012). 22344443 10.1126/science.1215904 PMC3338336

[b9] Gonzalez A., Cordomí A., Caltabiano G. & Pardo L. Impact of Helix Irregularities on Sequence Alignment and Homology Modeling of G Protein-Coupled Receptors. ChemBioChem 13, 1393–1399 (2012). 22761034 10.1002/cbic.201200189

[b10] Buch I., Harvey M. J., Giorgino T., Anderson D. P. & De Fabritiis G. High-Throughput All-Atom Molecular Dynamics Simulations Using Distributed Computing. J. Chem. Inf. Model. 50, 397–403 (2010). 20199097 10.1021/ci900455r

[b11] Dror R. O. . Structural basis for modulation of a G-protein-coupled receptor by allosteric drugs. Nature 503, 295–299 (2013). 24121438 10.1038/nature12595

[b12] Provasi D., Bortolato A. & Filizola M. Exploring Molecular Mechanisms of Ligand Recognition by Opioid Receptors with Metadynamics. Biochemistry (Mosc.) 48, 10020–10029 (2009). 10.1021/bi901494n PMC2764813 19785461

[b13] Hurst D. P. . A lipid pathway for ligand binding is necessary for a cannabinoid G protein-coupled receptor. J. Biol. Chem. 285, 17954–17964 (2010). 20220143 10.1074/jbc.M109.041590 PMC2878557

[b14] Doerr S. & De Fabritiis G. On-the-Fly Learning and Sampling of Ligand Binding by High-Throughput Molecular Simulations. J. Chem. Theory Comput. (2014), doi: 10.1021/ct400919u. 26580533

[b15] Kohlhoff K. J. . Cloud-based simulations on Google Exacycle reveal ligand modulation of GPCR activation pathways. Nat. Chem. 6, 15–21 (2014). 24345941 10.1038/nchem.1821 PMC3923464

[b16] Park J. H., Scheerer P., Hofmann K. P., Choe H.-W. & Ernst O. P. Crystal structure of the ligand-free G-protein-coupled receptor opsin. Nature 454, 183–187 (2008). 18563085 10.1038/nature07063

[b17] Hildebrand P. W. . A Ligand Channel through the G Protein Coupled Receptor Opsin. PLoS One 4, e4382 (2009). 19194506 10.1371/journal.pone.0004382 PMC2632885

[b18] Schmidtke P., Bidon-Chanal A., Luque F. J. & Barril X. MDpocket: open-source cavity detection and characterization on molecular dynamics trajectories. Bioinforma. Oxf. Engl. 27, 3276–3285 (2011). 10.1093/bioinformatics/btr550 21967761

[b19] González A., Perez-Acle T., Pardo L. & Deupi X. Molecular Basis of Ligand Dissociation in β-Adrenergic Receptors. PLoS One 6, e23815 (2011). 21915263 10.1371/journal.pone.0023815 PMC3168429

[b20] Bock A. . The allosteric vestibule of a seven transmembrane helical receptor controls G-protein coupling. Nat. Commun. 3, 1044 (2012). 22948826 10.1038/ncomms2028 PMC3658004

[b21] Freire E. Do enthalpy and entropy distinguish first in class from best in class? Drug Discov. Today 13, 869–874 (2008). 18703160 10.1016/j.drudis2008.07.005 PMC2581116

[b22] Böhm H.-J. & Klebe G. What Can We Learn from Molecular Recognition in Protein–Ligand Complexes for the Design of New Drugs? Angew. Chem. Int. Ed. Engl. 35, 2588–2614 (1996).

[b23] Stanley N., Esteban-Martín S. & De Fabritiis G. Kinetic modulation of a disordered protein domain by phosphorylation. Nat. Commun. 5, (2014). 10.1038/ncomms6272 25348080

[b24] Noé F. . Dynamical fingerprints for probing individual relaxation processes in biomolecular dynamics with simulations and kinetic experiments. Proc. Natl. Acad. Sci. 108, 4822–4827 (2011). 21368203 10.1073/pnas.1004646108 PMC3064371

[b25] Harrigan M. P., Shukla D. & Pande V. S. Conserve Water: A Method for the Analysis of Solvent in Molecular Dynamics. J. Chem. Theory Comput. (2015), doi: 10.1021/ct5010017. 26579759

[b26] Chrencik J. E. . Crystal Structure of Antagonist Bound Human Lysophosphatidic Acid Receptor 1. Cell 161, 1633–1643 (2015). 26091040 10.1016/j.cell.2015.06.002 PMC4476059

[b27] Parrill A. L. . Identification of Edg1 Receptor Residues That Recognize Sphingosine 1-Phosphate. J. Biol. Chem. 275, 39379–39384 (2000). 10982820 10.1074/jbc.M007680200

[b28] Nygaard R. . The Dynamic Process of β2-Adrenergic Receptor Activation. Cell 152, 532–542 (2013). 23374348 10.1016/j.cell.2013.01.008 PMC3586676

[b29] Perez-Aguilar J. M., Shan J., LeVine M. V., Khelashvili G. & Weinstein H. A Functional Selectivity Mechanism at the Serotonin-2A GPCR Involves Ligand-Dependent Conformations of Intracellular Loop 2. J. Am. Chem. Soc. 136, 16044–16054 (2014). 25314362 10.1021/ja508394x PMC4235374

[b30] Jo S., Lim J. B., Klauda J. B. & Im W. CHARMM-GUI Membrane Builder for Mixed Bilayers and Its Application to Yeast Membranes. Biophys. J. 97, 50–58 (2009). 19580743 10.1016/j.bpj.2009.04.013 PMC2711372

[b31] Humphrey W., Dalke A. & Schulten K. VMD: Visual molecular dynamics. J. Mol. Graph. 14, 33–38 (1996). 8744570 10.1016/0263-7855(96)00018-5

[b32] MacKerell . All-Atom Empirical Potential for Molecular Modeling and Dynamics Studies of Proteins†. J Phys Chem B 102, 3586–3616 (1998). 24889800 10.1021/jp973084f

[b33] Mackerell A. D., Feig M. & Brooks C. L. Extending the treatment of backbone energetics in protein force fields: Limitations of gas-phase quantum mechanics in reproducing protein conformational distributions in molecular dynamics simulations. J. Comput. Chem. 25, 1400–1415 (2004). 15185334 10.1002/jcc.20065

[b34] Vanommeslaeghe K., Raman E. P. & MacKerell A. D. Automation of the CHARMM General Force Field (CGenFF) II: Assignment of bonded parameters and partial atomic charges. J. Chem. Inf. Model. (2012), doi: 10.1021/ci3003649. PMC3528813 23145473

[b35] Vanommeslaeghe K. & MacKerell A. D. Automation of the CHARMM General Force Field (CGenFF) I: bond perception and atom typing. J. Chem. Inf. Model. 52, 3144–3154 (2012). 23146088 10.1021/ci300363c PMC3528824

[b36] Vanommeslaeghe K. . CHARMM general force field: A force field for drug-like molecules compatible with the CHARMM all-atom additive biological force fields. J. Comput. Chem. 31, 671–690 (2010). 19575467 10.1002/jcc.21367 PMC2888302

[b37] Yu W., He X., Vanommeslaeghe K. & MacKerell A. D. Extension of the CHARMM general force field to sulfonyl-containing compounds and its utility in biomolecular simulations. J. Comput. Chem. 33, 2451–2468 (2012). 22821581 10.1002/jcc.23067 PMC3477297

[b38] Harvey M. J., Giupponi G. & De Fabritiis G. ACEMD: Accelerating Biomolecular Dynamics in the Microsecond Time Scale. J. Chem. Theory Comput. 5, 1632–1639 (2009). 26609855 10.1021/ct9000685

[b39] Bowman G. R., Beauchamp K. A., Boxer G. & Pande V. S. Progress and challenges in the automated construction of Markov state models for full protein systems. J. Chem. Phys. 131, 124101 (2009). 19791846 10.1063/1.3216567 PMC2766407

[b40] Prinz J.-H. . Markov models of molecular kinetics: Generation and validation. J. Chem. Phys. 134, 174105–174105–23 (2011). 21548671 10.1063/1.3565032

[b41] Doerr S. & De Fabritiis G. *HTMD - Molecular modelling made simple* (2015) Available at: https://www.htmd.org/htmd/index.html (Accessed: 11th January 2016).

