# Supplementary material for: The pathway of ligand entry from the membrane bilayer to a lipid G protein-coupled receptor
Source: Sci Rep. 2016 Mar 4;6:22639. doi: 10.1038/srep22639 (PMC4778059; doi:10.1038/srep22639)
Supplement: Supplementary Information [file srep22639-s1.pdf]

The pathway of ligand entry from the membrane bilayer to a lipid G protein-coupled receptor

Supplementary Information

Nathaniel Stanley[a], Leonardo Pardo[b] and Gianni De Fabritiis[a,c]

[a]Computational Biophysics Laboratory (GRIB-IMIM), Universitat Pompeu Fabra, Barcelona Biomedical Research Park (PRBB), C/Doctor Aiguader 88, 08003 Barcelona, Spain.
[b]Laboratori de Medicina Computacional, Unitat de Bioestadística, Facultat de Medicina, Universitat Autònoma de Barcelona, 08193 Bellaterra, Spain.
[c]Institució Catalana de Recerca i Estudis Avançats, Passeig Lluis Companys 23, 08010 Barcelona, Spain

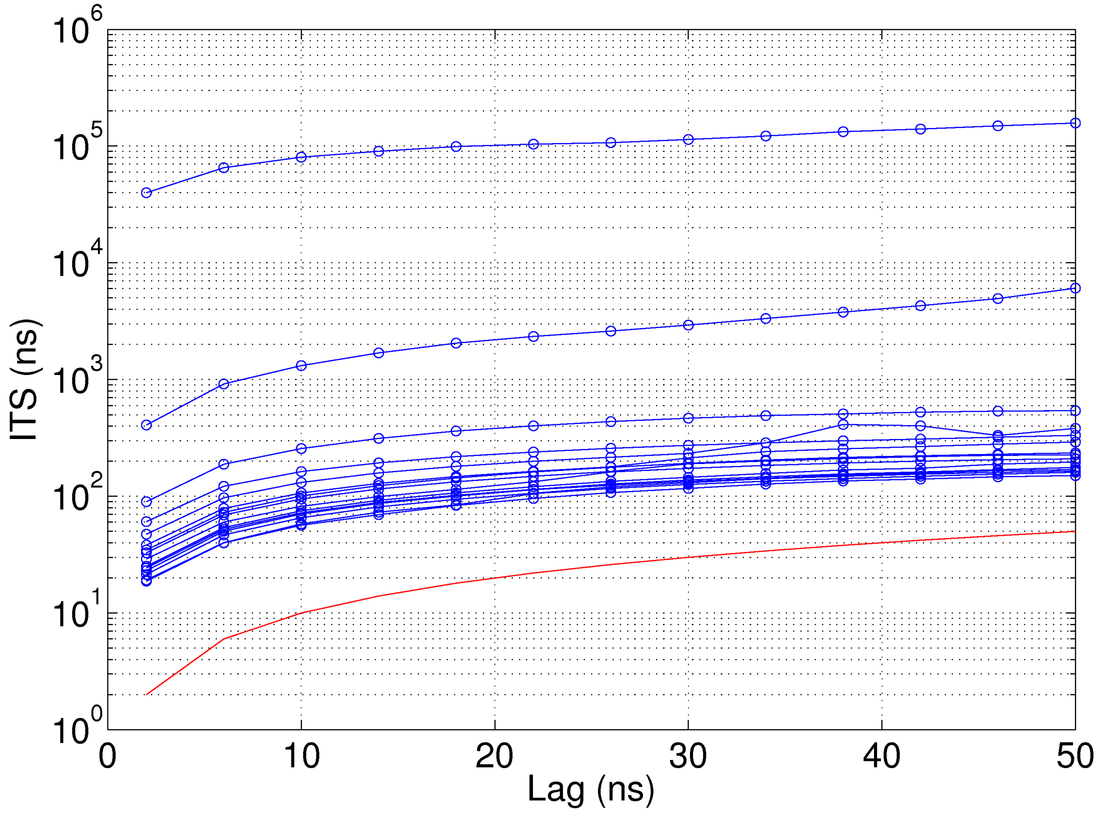

Supplementary Figure 1. Implied timescales of slowest processes: This plot shows the implied timescales (blue lines) of the slowest relaxation processes in the system as determined by Markov State Model analysis (see Methods). The highest line represents flipping of ML056 between the leaflets of the bilayer, and has a relaxation time of 100 µs. The second highest represents the bulk to bound transition, with a time between 1-10 µs. And the third line is the transition between intermediate (such as in Fig. 2b) and the bound state, at approximately 500 ns.

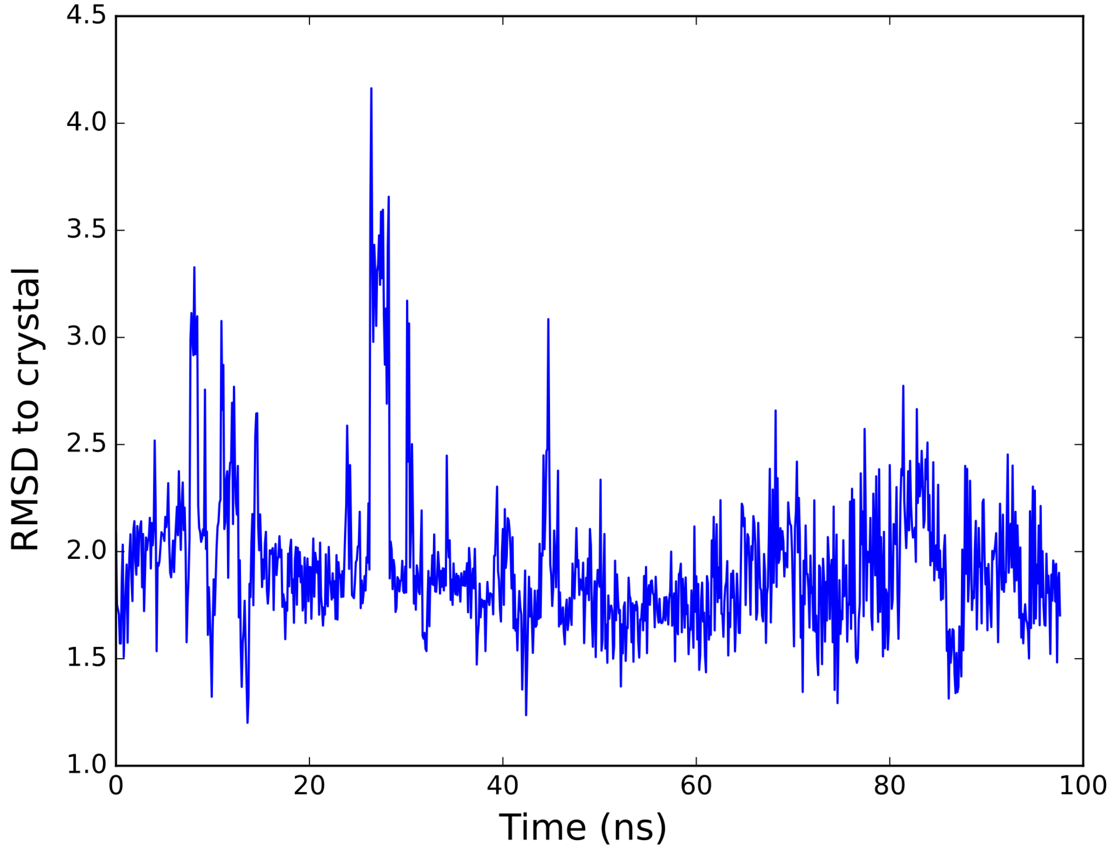

Supplementary Figure 2. Stability of the ligand when started from the bound pose: We assessed the stability of the bound pose by measuring the RMSD of the ligand compared to that of the ligand. Similar to simulations from the second round of respawning, the ligand remains 2Å RMSD to the crystal structure.

Supplementary Movie 1. The binding of ML056 to $S1P_1R$. $S1P_1R$ is shown as white cartoon structure, with residues that come within 5 angstroms of ML056 atoms shown transiently as cyan sticks. Total time shown is 1,156 nanoseconds.

Supplementary Movie 2. The binding of four ML056 ligands from different simulations, showing the increase in the degree of unfoldedness of the N-terminal helix as the ligands begin to interact. The ligands and their corresponding N-terminal helices are colored similarly, while the remaining part of the $S1P_1R$ is shown in white. Time shown is 913 nanoseconds.
